# A highly aqueous HPLC-PDA method for the assay of gabapentin and pregabalin: applications in pharmaceutical analysis, dissolution testing, and forensic screening

**DOI:** 10.3389/fchem.2026.1845390

**Published:** 2026-06-26

**Authors:** Fatimah M. Alamri, Taher Sahlabji, Yahya M. Alshehri, Ibrahim A. Al Othaim, Fahad S. Aldawasri, Abubakr M. Idris

**Affiliations:** 1 Department of Chemistry, College of Science, King Khalid University (KKU), Abha, Saudi Arabia; 2 Department of Chemistry, College of Science, University of Bisha (UB), Bisha, Saudi Arabia; 3 Saudi Food and Drug Authority (SFDA), Riyadh, Saudi Arabia

**Keywords:** derivatisation-free analysis, dissolution testing, forensic screening, gabapentinoids, green analytical chemistry, highly aqueous mobile phase, HPLC-PDA

## Abstract

**Introduction:**

Gabapentinoids, particularly pregabalin (PGL) and gabapentin (GBP), are widely used for neurological and psychiatric conditions but are increasingly linked to misuse and forensic challenges. Their high polarity, zwitterionic nature, and lack of strong chromophores present significant analytical challenges, often requiring derivatisation or specialised chromatographic systems. This study aimed to develop and validate a green, derivatisation-free HPLC-PDA method for the simultaneous determination of PGL and GBP in pharmaceutical formulations and seized samples.

**Methods:**

Chromatographic separation was achieved on a Hypersil GOLD™ C18 column (150 × 3 mm, 3 µm). An isocratic, highly aqueous mobile phase of water:methanol (96:4, v/v) was used at a flow rate of 0.7 mL/min. Detection was performed at 205 nm. Method optimisation was supported by a 2^3^ full factorial design. The method was validated in accordance with International Council for Harmonisation Q2(R2) guidelines, including linearity, accuracy, precision, specificity, robustness, and sensitivity.

**Results:**

The proposed method achieved effective separation without derivatisation, buffer systems, or specialised columns, while reducing organic solvent consumption compared to some reported methods. Calibration was linear over 5–60 μg/mL (r ≥ 0.9997). Accuracy ranged from 95%–103%. Intra- and inter-day precision (%RSD) values were below 5%. The limits of detection/quantification values were 0.31/1.03 μg/mL for PGL and 0.25/0.85 μg/mL for GBP. Dissolution studies confirmed rapid drug release meeting pharmacopoeial criteria. Application to seized samples revealed PGL content exceeding labelled values (106%–110%), while no peak corresponding to GBP was observed under the current HPLC-PDA conditions.

**Conclusion:**

The proposed method provides a simple, reliable, and environmentally favourable analytical approach for the simultaneous determination of gabapentinoids without complex sample preparation. Its successful application to pharmaceutical analysis, dissolution testing, and forensic samples highlights its suitability for routine laboratory use and regulatory monitoring.

## Graphical Abstract

**Figure:**
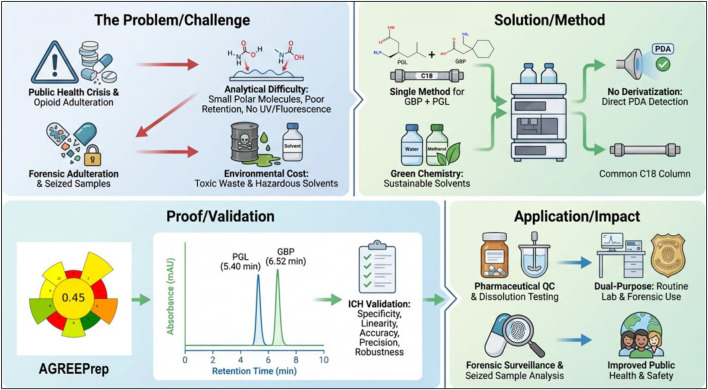

## Highlights

- A derivatisation-free HPLC-PDA method was developed for simultaneous quantification of gabapentin and pregabalin.
- The method employs a 96% aqueous mobile phase, reducing organic solvent consumption relative to selected reported methods.
- AGREEPrep score (0.45) indicates improved greenness compared to reported methods.
- Dissolution profiles confirmed rapid drug release meeting pharmacopoeial criteria.
- Forensic application revealed seized pregabalin samples exceeding labelled content by 6%–10%.
- No peak corresponding to gabapentin was observed under the current HPLC-PDA conditions in seized samples.

## 1 Introduction

**FIGURE 1:**
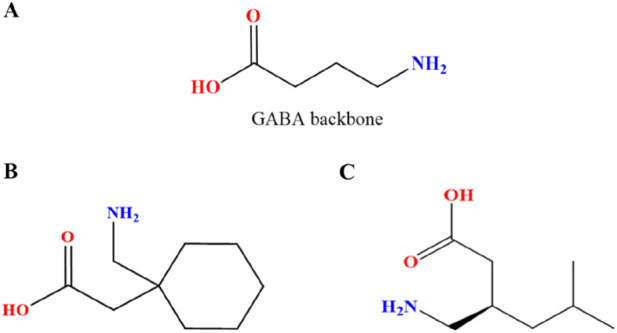
Chemical structures of **(A)** γ-aminobutyric acid (gabapentinoids), **(B)** gabapentin [1-(aminomethyl)cyclohexaneacetic acid] and **(C)** the (S)-enantiomer of pregabalin [(S)-(+)-3-(aminomethyl)-5-methylhexanoic acid].

Gabapentinoids, mainly gabapentin (GBP) and pregabalin (PGL), are a class of medications that are structural analogs of the inhibitory neurotransmitter γ-aminobutyric acid (Figure 1A) (Nahar et al., 2017; Ananda et al., 2003; Taylor et al., 1998; Rheims et al., 2015). They exert their primary effect through high-affinity binding to the alpha-2-delta (α2δ) subunit of voltage-gated calcium channels, reducing calcium influx and inhibiting excitatory neurotransmitter release (Kendall et al., 2023; Temmermand et al., 2022). GBP and PGL were originally designed as anticonvulsants to treat epilepsy and neuropathic pain. Today, their use has expanded to include generalised anxiety disorder and, in some regions, off-label applications for alcoholism and restless leg syndrome (Nahar et al., 2017; De La Vega et al., 2019).

In detail, GBP is formally known as 1-(aminomethyl)cyclohexaneacetic acid, with a molecular formula of C_9_H_17_NO_2_ and a molecular weight of 171.24 g/mol (Ibers, 2001) (Figure 1B). In 1993, the U.S. Food and Drug Administration (FDA) approved GBP as an adjunctive therapy for partial-onset seizures and later for postherpetic neuralgia (Yasaei et al., 2025; Peckham et al., 2018). A related drug, PGL, is named (S)-3-(aminomethyl)-5-methylhexanoic acid with the molecular formula C_8_H_17_NO_2_ and a molecular weight of 159.23 g/mol (Rheims et al., 2015) (Figure 1C). It was approved in 2004 by the FDA in the U.S. for the treatment of neuropathic pain, fibromyalgia, generalised anxiety disorder, and as an adjunct therapy for partial-onset seizures (Tassone et al., 2007).

Despite their structural similarity, PGL exhibits higher bioavailability and a faster onset of action than GBP, which may contribute to its greater abuse liability (Mayoral et al., 2025). However, despite their therapeutic value, GBP and PGL present a dual challenge of common adverse effects, including dizziness, somnolence, and peripheral oedema, together with a growing potential for misuse and abuse. GBP can induce euphoria at elevated doses and is misused to potentiate opioids (Peckham et al., 2018; Tassone et al., 2007), whereas PGL shows greater abuse liability (Schifano, 2014). Furthermore, the concomitant use of these gabapentinoids with other central nervous system (CNS) depressants, particularly opioids, creates a dangerous synergy that markedly increases the risk of life-threatening respiratory depression and fatal overdose. These concerns emphasise the essential role of robust analytical methods in therapeutic drug monitoring and forensic investigation (Goins et al., 2021; Straube, 2024; Evoy et al., 2017).

In addition, the concurrent use of gabapentinoids represents a significant and growing public health threat. Forensic toxicology analyses of seized materials have confirmed the illicit practice of combining these drugs for abusive purposes, as demonstrated in our previous study (Alamri et al., 2025), which identified GBP as an adulterant in seized PGL capsules. This combination amplifies sedation, dizziness, and cognitive impairment (Athavale and Murnion, 2023). This practice underscores the urgent need for continued monitoring of the illicit drug supply and public health warnings regarding the dangers of polysubstance gabapentinoid abuse, as well as the necessity for analytical methods capable of simultaneously quantifying both gabapentinoids to effectively assess product quality.

In another context, GBP and PGL constitute a subgroup of zwitterionic antiepileptic drugs characterised by their analogous chemical structures, each featuring both amino and carboxylic acid functional groups (Figure 1). This structural similarity directly translates to comparable physicochemical properties. Both compounds present as white to off-white crystalline solids and exhibit nearly identical dissociation constants, with GBP demonstrating pKa values of 3.7 and 10.7, while PGL shows corresponding values of 4.2 and 10.6. Both compounds exhibit similar dissociation constants and solubility profiles, being freely soluble in water due to their zwitterionic nature (Kostić et al., 2014). From an analytical chemistry perspective, in addition to their structural similarities, GBP and PGL present significant challenges for chromatographic analysis due to their small size, high polarity, and zwitterionic nature at physiological pH, which result in poor retention on traditional reversed-phase HPLC columns (Kostić et al., 2014; Mahgoub et al., 2025). Furthermore, the absence of strong chromophores or native fluorophores has led many reported methods to rely on pre-column derivatisation for fluorescence detection (Ulu and Kel, 2011; Douša et al., 2010). These derivatisation procedures increase analytical complexity and may limit high-throughput analysis, while gas chromatography (GC) methods face similar challenges due to extensive sample preparation requirements (Mudiam et al., 2012; Hložek et al., 2016; Hitchcock and Marginean, 2019). Moreover, many reported liquid chromatography methods that avoid derivatisation do so with high environmental and operational costs. They often employ mobile phases with high organic solvent content or require specially buffered systems to control ionisation and achieve adequate retention (Goday et al., 2018; Khanage et al., 2014; Udaykumar Rao et al., 2009; Vardhman Chemtech Ltd et al., 2009). These conditions increase the consumption of hazardous solvents, generate substantial toxic waste, and raise long-term operational costs, conflicting with the principles of green analytical chemistry.

To address these limitations, this study aimed to develop and validate a green, derivatisation-free high-performance liquid chromatography with photodiode array detection **(**HPLC-PDA) method for the simultaneous quantification of GBP and PGL, with applications in pharmaceutical assay, dissolution testing, and forensic analysis of seized samples. Despite numerous reported methods for their determination, most rely on derivatisation, specialised stationary phases, or mobile phases containing high proportions of organic solvents to achieve adequate retention and detection sensitivity. In contrast, the proposed method employs a highly aqueous mobile phase (96% water) and a conventional C18 column to enable effective separation and quantification of both analytes. This demonstrates that sufficient retention, resolution, and sensitivity can be achieved for these polar, zwitterionic compounds under predominantly aqueous conditions without the need for buffer systems or specialised columns. Beyond method development, the present work offers several broader contributions that were not part of our previous study (Alamri et al., 2025): (i) simultaneous determination of both gabapentinoids, (ii) application to dissolution testing of pharmaceutical capsules, (iii) quantitative greenness assessment using AGREEPrep, and (iv) full method validation according to International Council for Harmonisation **(**ICH) Q2(R2) guidelines. The method therefore provides a simplified and environmentally favourable alternative for routine analysis while addressing key limitations of previously reported approaches.

## 2 Experimental

### 2.1 Chemicals and reagents

A GBP reference standard (purity 99.8%) was obtained from Pfizer Inc., (United States), while a PGL reference standard (purity ≥99.9%) was acquired from HPC Standards GmbH (Germany). HPLC-grade methanol (≥99.9%) was purchased from Sigma-Aldrich (Germany). Hydrochloric acid (37%) was obtained from Merck (Darmstadt, Germany). Ultrapure water (18.2 MΩ·cm) was produced using a Duo™ water purification system supplied by Avidity Science™ (United States).

### 2.2 Instrumentation and chromatographic conditions

Chromatographic analysis was performed using a Prominence LC-20AD HPLC system from Shimadzu Corporation (Japan). The system was equipped with a photodiode array (PDA) detector. Separation was achieved using a Thermo Scientific Hypersil GOLD™ analytical column (150 × 3 mm, 3 μm) maintained at 25 °C. For dissolution profile studies, a Sotax AT 7 Smart dissolution apparatus (Sotax AG, Basel, Switzerland) was employed using Apparatus 2 (paddle) at 50 revolutions per minute. The dissolution medium was maintained at 37.0 °C ± 0.5 °C throughout the testing.

The analysis employed isocratic elution with a mobile phase composed of purified water:methanol (96:4, v/v) delivered at a constant flow rate of 0.7 mL/min. The total run time was 10 min with an injection volume of 10 μL. The autosampler temperature was maintained at 5 °C to ensure sample stability. Detection was performed at a wavelength of 205 nm.

### 2.3 Multivariate optimisation using design of experiments

To validate and refine the optimal conditions identified through sequential univariate optimisation, a multivariate experimental design method was employed. A full factorial design was constructed to systematically evaluate the interactions and combined effects of the three most critical parameters: methanol percentage (4% and 6%), injection volume (10 and 20 µL), and flow rate (0.7 and 1.5 mL/min). The chromatographic resolution (R) between PGL and GBP peaks was selected as the critical response.

### 2.4 Standard solution preparation

Primary stock solutions of GBP and PGL (1,000 μg/mL each) were prepared separately by dissolving 2 mg of each reference standard in 2 mL of diluent matching the mobile phase composition to ensure chromatographic consistency. A mixed standard solution containing both GBP and PGL (100 μg/mL each) was prepared by combining 0.5 mL aliquots from each primary stock solution and diluting to a final volume of 5 mL with diluent. According to the ICH guidelines for method validation (ICH, 2023), calibration standards were prepared by serial dilution of the mixed standard solution to yield concentrations of 5, 10, 20, 30, 40, 50, and 60 μg/mL for both analytes. All solutions were stored at 4 °C and protected from light.

### 2.5 Sampling and sample preparation

Pharmaceutical capsules containing GBP (400 mg) and PGL (50 mg and 150 mg), which were sourced from the Saudi Food and Drug Authority (SFDA), were used for analytical validation and dissolution profiling. Suspected adulterated gabapentinoid products were also acquired as seized specimens from the SFDA’s Medicines Control Division. Capsules were individually processed to obtain stock solutions. Each capsule was dissolved in water:methanol (96:4) diluent, with PGL diluted to 50 mL and GBP to 100 mL. Aliquots were subsequently diluted to achieve final working concentrations of 35 μg/mL for both analytes. All solutions were prepared in triplicate, vigorously shaken for 5 min, and filtered through 0.45-μm nylon membrane filters prior to analysis.

Homogenised seized powder samples (10 mg) were accurately weighed and dissolved in 10 mL of water:methanol (96:4) diluent to yield 1 mg/mL stock solutions. After 15 min of ultrasonic agitation and filtration through 0.45-μm nylon membranes, appropriate aliquots were diluted to achieve final working concentrations of 35 μg/mL for chromatographic analysis.

### 2.6 Method validation

The optimised HPLC-PDA method was validated according to ICH guideline Q2(R2) (ICH, 2023). Specificity was confirmed through the analysis of blank diluent and placebo solutions, ensuring that no interfering peaks appeared at the retention times of the target analytes. In addition to individual injections of GBP and PGL, reference standards were used to confirm their retention times. Carry-over was evaluated by injecting blank solvent (water:methanol, 96:4 v/v) immediately following the highest calibration standard (60 μg/mL). Acceptance criteria were set such that the signal in blank injections was <20% of the LOQ for specificity and ≤5% of the upper calibration limit for carry-over assessment.

Linearity was evaluated using seven calibration standards across the concentration range of 5–60 μg/mL for both GBP and PGL. Each concentration level was prepared and analysed in triplicate (n = 3). Peak area responses were plotted against corresponding concentrations. Linearity was assessed through calculation of the correlation coefficient (r) and visual examination of residual plots. The limits of detection (LOD) and quantification (LOQ) were determined from six replicate injections of the lowest calibration standard (5 μg/mL). LOD and LOQ were calculated based on the standard deviation (SD) of the response and slope (S) of the calibration curve using Equations 1, 2, respectively.

$$LOD=3.3\;\left(SD/S\right)$$ (1)

$$LOQ=10\;\left(SD/S\right)$$ (2)

Recovery was assessed in terms of the percentage recovery of known amounts of analytes spiked into the blank matrix, and it was calculated using Equation 3. Precision was assessed through repeatability and intermediate precision studies. It is expressed as relative standard deviation (RSD%) of peak areas using Equation 4. System repeatability was tested by six replicate injections of a 40 μg/mL standard from a single HPLC vial of each analyte. Method repeatability (intra-day precision) was established by analysing blank matrix samples spiked with GBP and PGL at three concentration levels (10, 40, and 60 μg/mL), prepared in triplicate and analysed within one run. Intermediate precision (inter-day precision) was examined by analysing three concentration levels (10, 40, and 60 μg/mL) across three different days by two analysts, with three independent preparations for each concentration.

$$\%Recovery=\left(\frac{Measured\;value}{Theoretical\;value}\right)\times 100$$ (3)

$$\%RSD=\left(\frac{SD\;of\;peak\;area}{Mean\;peak\;area}\right)\times 100$$ (4)

The robustness of the method was evaluated through solution stability studies and deliberate variations of HPLC parameters. Solution stability was assessed at two concentration levels (5 and 40 μg/mL) under autosampler conditions at 20 °C. Duplicate preparations at each level were analysed immediately after preparation and at 3, 6, 24, 48, and 72-h intervals. Method robustness was further tested by introducing deliberate variations to key HPLC parameters, including flow rate (±0.1 mL/min), column temperature (±2 °C), and detection wavelength (±2 nm). All solutions were prepared in duplicate, with single injections performed under each modified condition.

### 2.7 Dissolution

Dissolution testing was performed in accordance with the British Pharmacopoeia (BP) monograph for GBP and PGL Capsules (British Pharmacopoeia Commission, 2025a; British Pharmacopoeia Commission, 2025b). A calibrated dissolution Apparatus 2 (paddle) was operated at 50 revolutions per min. Nine hundred millilitres of freshly prepared hydrochloric acid solution (0.1 mol/L for GBP and 0.06 mol/L for PGL) were used as the dissolution medium for each respective drug, and the temperature was maintained at 37 °C ± 0.5 °C.

For each drug product, six capsules were tested individually. Samples (2 mL) were automatically withdrawn from each vessel at 5, 10, 15, 20, 30, 40, and 60-min intervals. Each sample was immediately filtered through a 0.45 μm nylon membrane filter. The filtered samples were analysed using the validated HPLC-PDA method.

The concentration of GBP and PGL in each dissolution sample was determined using the validated HPLC-PDA method. Quantification was achieved by interpolation from a linear calibration curve of peak area versus concentration, prepared daily using certified standards. The percentage of drug dissolved was calculated using Equation 5:

$$\%Dissolution=\frac{C\times 10\times 900}{Dose}\times 100$$ (5)

where C is the HPLC-derived concentration (µg/mL), 10 represents the dilution factor, 900 represents the volume of the dissolution medium (mL), and dose is the capsule strength (150,000 µg for PGL or 400,000 µg for GBP).

The pH of the dissolution medium was approximately 1.2 for PGL (0.06 mol/L HCl) and 1.0 for GBP (0.1 mol/L HCl). Sink conditions were maintained throughout the dissolution test. The solubility of both PGL and GBP in the respective acidic media exceeded three times the highest concentration tested (166.7 μg/mL for PGL and 444.4 μg/mL for GBP). This complied with the requirements of the BP for dissolution testing (Pharmacopoeia Commission, 2025).

### 2.8 Experimental design optimization

2^3^ full factorial design was proposed after scanning all conditions presumed to be considerably controlling the resolution using the univariate approach. The 2^3^ multivariate approach was carried out for methanol% (MeOH%), injection volume, and flow rate. The main effect and interaction effects were calculated using Equation 6; where *y(+1)* is the response value at the maximum level, *y(-1)* is the response value at the minimum level, and *n* is the number of variables at the same level.

$$E_{f}=\left(\;\frac{\sum y\left(+1\right)}{n}-\frac{\sum y\left(-1\right)}{n}\;\right)$$ (6)

All experiments were performed in a single run without replicates. Therefore, pure error cannot be estimated, and ANOVA with p-values is not reported. Factor significance is assessed based on calculated effect magnitudes and the Pareto chart, which is an accepted approach for screening factorial designs when replicate experiments are not feasible.

## 3 Results and discussion

### 3.1 Method optimisation

Due to their polarity, chromatographic optimisation was required to achieve adequate retention. These characteristics lead to poor retention and resolution on standard reversed-phase columns. Therefore, method optimisation was initiated with a comprehensive evaluation of stationary phases to tackle the challenge of poor retention for PGL and GBP. Initial screening used standard C_18_ columns (150 × 2 mm and 150 × 4.6 mm, 3 µm), which did not provide sufficient resolution. A notable improvement was observed when switching to a polar-embedded phase. Hypersil GOLD™ column (150 × 3 mm, 3 µm) provided better peak shapes and resolution through balanced retention mechanisms. Hypersil GOLD™ column’s high-purity silica and dense, double-endcapped C18 bonding features minimum silanol interactions, a common cause of peak tailing for polar basic compounds. Furthermore, its wide pH stability allows for robust method development, ensuring the phase integrity when testing various aqueous-organic mobile phase combinations. For mobile phase optimisation, acetonitrile-based and mixed organic modifier (MeOH:acetonitrile(CAN)) led to co-elution or suboptimal resolution. Notably, buffered systems such as 0.01 M potassium dihydrogen phosphate (pH 3), 0.003 M ammonium formate (pH 3), and 0.02 M sodium acetate (pH 5) resulted in reduced analyte response. This effect is likely due to changes in ionisation state and reduced UV absorbance, indicating their unsuitability. In contrast, a simple water:methanol (96:4, v/v) mixture provided improved chromatographic performance, with enhanced separation efficiency, peak shape, and reproducibility. The organic solvent ratio was systematically evaluated. A minimal methanol concentration of 4% in the aqueous mobile phase yielded superior resolution compared with higher percentages (5% and 6%), likely due to enhanced interaction of the polar analytes with the stationary phase. As a result, combining the Hypersil GOLD™ column with this straightforward, isocratic, and unbuffered mobile phase was selected as the optimal chromatographic condition based on resolution, peak shape, and reproducibility for this purpose. Subsequently, the sample dilution solvent was aligned with the mobile phase composition to further improve the method; changing from water:ACN (90:10, v/v) to water:MeOH (96:4, v/v) significantly enhanced the separation, underscoring the importance of solvent-strength matching to prevent on-column focusing issues and peak distortion.

The second stage of method optimisation focused on fine-tuning critical chromatographic parameters to achieve maximum sensitivity and resolution. Wavelength selection was conducted by scanning from 190 nm to 400 nm. 205 nm was selected as the optimum wavelength as it provided the highest absorption and thus optimal sensitivity for the analyte mixture, despite being a non-selective wavelength. However, selectivity was confirmed through analysis of real samples and blanks, as detailed in Section 2.6. Flow rate optimisation revealed that a slower flow rate of 0.7 mL/min, selected after evaluating a range from 0.7 to 1.5 mL/min, significantly improved chromatographic separation by allowing for more efficient mass transfer. This lower flow rate also provided the practical benefit of reducing mobile phase consumption. Different injection volumes (10, 15, and 20 µL) were evaluated. The 10 µL volume was selected as optimal because it yielded sharper peaks and better resolution, while also reducing solvent consumption for a more environmentally friendly method. Finally, the influence of column oven temperature was investigated across a range of 30 °C–40 °C. The primary role of temperature in this method is to ensure baseline stability, a fundamental prerequisite for reliable quantification. The tested higher temperatures (30 °C–40 °C) were found to induce baseline instability. This effect is likely caused by an increased propensity for bubble formation in the highly aqueous mobile phase and a disruption of the sensitive thermal equilibrium required for the analysis of these polar compounds. As a result, a lower ambient temperature of 25 °C is recommended as the optimal condition, as it preserves the method’s stability and ensures the integrity of the chromatographic separation. Using a low-organic, high-aqueous mobile phase at a reduced flow rate achieved our analytical goals of peak sharpness and baseline resolution.

**FIGURE 2:**
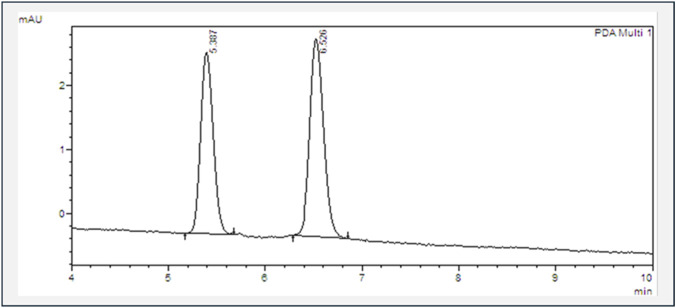
HPLC-PDA chromatogram of a mixed standard solution (40 μg/mL); chromatographic conditions: Hypersil GOLD™ C18 (150 × 3 mm, 3 µm); mobile phase: water:MeOH (96:4, v/v); flow rate: 0.7 mL/min, column temperature: 25 °C, detection: 205 nm.

In addition, Supplementary Table S1 (Supplementary Material) shows the experimental design results. The table demonstrates that the highest resolution (R = 3.303) was achieved under the specific combination of 4% methanol, a 10 µL injection volume, and a 0.7 mL/min flow rate (experiment 1) (Figure 2). This combination outperformed all other parameter sets. Crucially, this multivariate optimum aligns perfectly with the optimal levels determined in the earlier univariate optimisation. The marked drop in resolution observed at higher flow rates (1.5 mL/min) or higher organic modifier percentages (6%) across all experiments confirms the initial univariate conclusions and underscores the robustness of the final method parameters.

**FIGURE 3:**
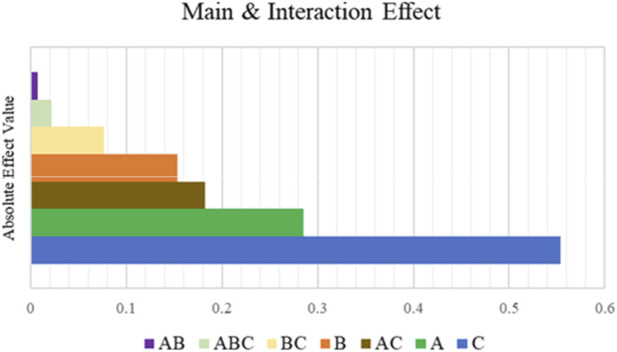
Pareto chart of standardised effects from the 2^3^ full factorial design for chromatographic resolution; A: MeOH%, B: injection volume, C: flow rate, AB: interaction between MeOH% and injection volume, AC: interaction between MeOH% and flow rate, BC: interaction between injection volume and flow rate, and ABC: three-way interaction among all factors.

The codes for the main and interaction factors are compiled in Supplementary Table S2, and the results are shown in Supplementary Table S3 and Figure 3. Based on effect magnitudes (Supplementary Table S3; Figure 3), flow rate (C) had the largest influence on resolution (effect = −0.554), followed by methanol percentage (A, effect = −0.285) and injection volume (B, effect = −0.153). This indicates that higher flow rates compromise separation efficiency more strongly than the other factors. Among interactions, only the MeOH%-flow rate interaction (AC) showed a notable positive effect (+0.182), suggesting that the impact of methanol content is modulated by flow rate.

Notably, the retention and separation of GBP and PGL under highly aqueous conditions (96% water) can be rationalised based on their physicochemical properties and interactions with the stationary phase. Both analytes are zwitterionic with limited intrinsic hydrophobicity. However, structural differences, particularly the cyclohexyl group in GBP compared to the isobutyl group in PGL, contribute to differences in hydrophobic interactions with the C18 phase. Under highly aqueous mobile phase conditions, the reduced elution strength enhances analyte-stationary phase interactions, enabling measurable retention despite their polarity. Partial ionisation of the analytes may further contribute to secondary interactions, including hydrogen bonding and weak electrostatic interactions with residual silanol groups. The absence of buffer systems may further improve analyte response by avoiding suppression of UV absorbance and minimising ion-pairing effects that could otherwise affect retention and sensitivity. The combination of low organic solvent content and controlled chromatographic conditions therefore supports effective separation without the need for derivatisation or alternative chromatographic modes. These findings indicate that highly aqueous reversed-phase systems can be effectively applied to the analysis of polar zwitterionic compounds when subtle structural differences and interaction mechanisms are appropriately exploited.

### 3.2 Method validation

The method validation demonstrated excellent specificity, confirmed through the analysis of blank diluent and placebo solutions, which showed no interfering peaks at the retention times of the target analytes (PGL: 5.40 min; GBP: 6.52 min). An additional selectivity assessment was performed using real samples. The signal in all blank injections was confirmed to be below 20% of the LOQ response, corresponding to <0.21 μg/mL for PGL and <0.17 μg/mL for GBP. This fulfilled the ICH Q2(R2) requirement for demonstrating no interference (ICH, 2023). Forced degradation and peak purity studies were not performed in this study. Therefore, the method is validated for the specific matrices tested (pharmaceutical capsules and seized forensic samples) rather than for comprehensive stability-indicating purposes. For routine forensic screening, the observed absence of co-eluting peaks in authentic samples supports method suitability, provided that negative findings are interpreted conservatively. Furthermore, carry-over was evaluated by injecting a blank solvent immediately after the highest calibration standard (60 μg/mL). The residual signal corresponded to less than 5% of the response at the upper calibration level, meeting the acceptance criterion. This result demonstrates that no significant carryover was observed, confirming selectivityfor the target analytes and that carry-over does not impact quantification accuracy. This finding confirms the method’s reliability for analysing both pharmaceutical and seized samples in accordance with ICH Q2(R2) principles.

**FIGURE 4:**
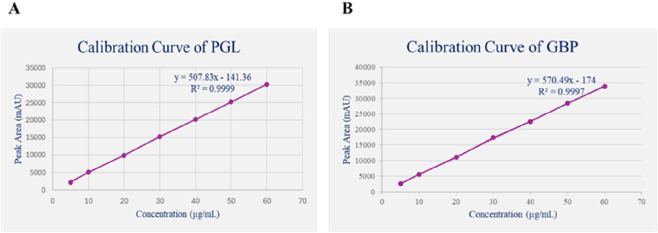
Calibration curves of **(A)** pregabalin and **(B)** gabapentin; chromatographic conditions: Hypersil GOLD™ C18 (150 × 3 mm, 3 µm); mobile phase composition water:MeOH (96:4, v/v); flow rate 0.7 mL/min, column temperature 25 °C, detection 205 nm.

The linearity of the method was successfully demonstrated for both PGL and GBP over a wide concentration range of 5–60 μg/mL (Figures 4A,B). The linear regression parameters are compiled in Table 1. The correlation coefficients were ≥0.9997 for both analytes, confirming a strong linear relationship between concentration and detector response. For PGL, the linear least-squares regression analysis yielded a slope of 507.83 and an intercept of −141.36. The relatively high slope value indicates good sensitivity and a strong response to concentration variations. The SD of the slope was 2.38, representing only 0.47% of the slope value and reflecting excellent precision in the calibration. Similarly, the SD of the intercept was 86.07, while the standard error of estimate was 119.70, demonstrating minimal scatter of data points around the regression line. For GBP, the regression analysis showed a slope of 570.49 and an intercept of −174.00. The SD of the slope was 4.59, representing 0.80% of slope value, and the SD of the intercept was 165.58. The standard error of estimate was 230.28, indicating slightly greater variability compared to PGL but still within acceptable limits for analytical method validation (Table 1). The observed negative Y-intercepts were statistically present but negligible relative to the analytical response at the upper calibration level. They represented less than 0.6% of the detector response at the upper limit of quantification (ULOQ). Consequently, they are not expected to introduce significant bias in quantitative results, and the linear model remains appropriate for accurate quantification across the validated range. The low standard errors and minimal variability in slope and intercept values for both analytes confirm the adequacy of the linear regression model and the reliability of the method for quantitative analysis of both PGL and GBP in pharmaceutical formulations and seized samples within the validated concentration range.

**TABLE 1:** Linear regression parameters for the HPLC-PDA determination of pregabalin and gabapentin.

Parameter	Pregabalin	Gabapentin
Concentration range (μg/mL)	5–60	5–60
Correlation coefficient	0.9999	0.9997
Slope	507.83	570.49
Standard deviation of slope	2.38	4.59
Intercept	−141.36	-174.00
Standard deviation of intercept	86.07	165.58
Standard error of estimate	119.70	230.28
Number of calibration points	7	7
Replicates per point	3	3

On the other side, the method demonstrated excellent sensitivity, with LOD and LOQ values of 0.31 and 1.03 μg/mL for PGL, and 0.25 and 0.85 μg/mL for GBP, respectively. These limits are particularly suitable for detecting low-level adulteration in forensic samples and for dissolution testing where analytes are highly diluted. Therefore, the results confirm the method’s suitability for both pharmaceutical analysis and forensic applications. The slightly better sensitivity for GBP (17.5% lower LOQ) corresponds with its higher response slope observed in linearity studies. Furthermore, the consistent 3:10 LOD-to-LOQ ratio for both analytes validates the calculation methodology and underscores uniform performance across the target compounds.

Accuracy, determined as percentage recovery, was excellent for both analytes, falling within the predefined acceptable ranges of 95%–103% for PGL and 95%–102% for GBP. Table 2 shows the precision results in terms of repeatability and intermediate precision. The results demonstrate good repeatability, with all %RSD values below 2.0%, and acceptable intermediate precision with all %RSD values below 5.0%, confirming the method’s robustness against inter-day and inter-analyst variations. The performance of both analytes is fully compliant with standard regulatory criteria, validating the method for their simultaneous quantification. This finding supports that the proposed method is accurate, precise, and fit-for-purpose.

**TABLE 2:** Precision data for the determination of pregabalin and gabapentin in pharmaceutical formulations: Method repeatability (intra-day) and intermediate precision (inter-day) expressed as relative standard deviation (%RSD) at three concentration levels (n = 3).

Analyte	Concentration µg/mL	Method repeatability (RSD%)	Intermediate precision (RSD%)
Pregabalin	10	1.91	4.87
	40	1.19	3.12
	60	1.37	4.23
Gabapentin	10	1.68	3.63
	40	1.78	4.45
	60	1.33	1.58

The method established acceptable robustness and stability. As shown in Tables 3, 4, both analytes showed excellent solution stability over 72 h, with %RSD values remaining below 3.0% at both concentration levels. Furthermore, under all deliberately varied chromatographic conditions, the %RSD for peak areas of both PGL and GBP was found to be within the accepted limit of 3.0% for the 5 and 40 μg/mL concentrations. This limit aligns with standard validation practice for robustness and stability, derived from the precision requirements of ICH Q2(R2) (ICH, 2023), illustrating the method’s reliability for its intended use.

**TABLE 3:** Solution stability data for pregabalin and gabapentin under autosampler storage conditions (20 °C) over 72 h.

Analyte	Concentration (µg/mL)	%RSD at different time points				Linear regression	
		0 h	3 h	24 h	72 h	Slope (%/h)	p-value
Pregabalin	5	1.03	1.24	2.55	2.59	0.02	0.18
	40	0.49	0.09	0.29	0.25	−0.00	0.85
Gabapentin	5	1.19	0.78	2.76	1.81	0.01	0.57
	40	0.70	0.90	1.29	0.02	−0.01	0.30

**TABLE 4:** Robustness evaluation: impact of deliberate HPLC parameter variations on method precision.

Analyte	C (µg/mL)	Standard condition (%RSD)	Altered condition (%RSD)					
			Flow rate (±0.1 mL/min)		Column T (±2 °C)		Detection wavelength (±2 nm)	
			Flow rate (+)	Flow rate (−)	Column T (+)	Column T (−)	Wavelength (+)	Wavelength (−)
Pregabalin	5	0.10	1.71	1.94	2.11	2.38	0.26	1.77
	40	0.12	0.03	0.01	2.08	0.41	1.09	0.61
Gabapentin	5	1.07	2.57	0.56	0.65	2.75	2.00	2.02
	40	1.25	1.96	1.38	0.93	0.22	0.85	1.31

Statistical evaluation using linear regression analysis (Table 3) objectively confirmed the absence of any significant time-dependent trend for all stability conditions. The slopes ranged from −0.01 to +0.02% h^-1^, with all p-values >0.05, exceeding the 0.05 significance threshold; there is no statistical evidence of degradation or instability over the 72 h, confirming method robustness.

### 3.3 Application of the method to pharmaceutical formulation analysis

**FIGURE 5:**
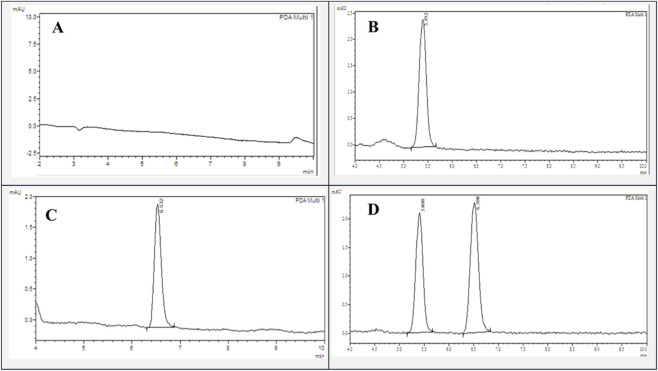
Representative HPLC-PDA chromatograms (λ = 205 nm) of **(A)** blank diluent, **(B)** pregabalin capsule formulation, **(C)** gabapentin capsule formulation, and **(D)** combined sample (pregabalin and gabapentin); chromatographic conditions: stationary phase Hypersil GOLD™ C18 (150 mm × 3 mm, 3 μm); mobile phase water: methanol (96:4, v/v); flow rate 0.7 mL/min; column temperature 25 °C.

The developed HPLC-PDA method was successfully applied to the analysis of PGL and GBP in pharmaceutical capsule formulations. As shown in the representative chromatograms (Figures 5A–D), the blank diluent (Figure 5A) exhibited no interfering peaks at the retention times of PGL (5.40 min) or GBP (6.52 min), confirming that under the validated conditions, no interfering peaks from excipients or matrix components were observed. The method exhibited excellent chromatographic performance, successfully resolving PGL and GBP at 35 μg/mL (Figures 5B,C). This efficacy was further validated by the accurate quantification of both analytes in a laboratory-prepared mixture (Figure 5D), with results matching the expected nominal concentrations, consistent with the data presented in Table 5. This outcome confirms the method’s accuracy and its capability for the simultaneous determination of both compounds in a mixture. The absence of significant interference at the respective retention times further emphasises the high specificity of the method. Overall, these results confirm the suitability of the method for routine assay of pharmaceutical formulations.

**TABLE 5:** Assay results for the determination of pregabalin and gabapentin in a pharmaceutical capsule formulation.

Analyte	Potency (mg/capsule)	Nominal concentration (µg/mL)	Measured concentration (µg/mL)	RSD%	Accuracy (%)
Pregabalin	50	35	35.81	0.21	102.32
Gabapentin	400	35	34.55	0.10	98.72

### 3.4 Application of the method to seized samples analysis

**FIGURE 6:**
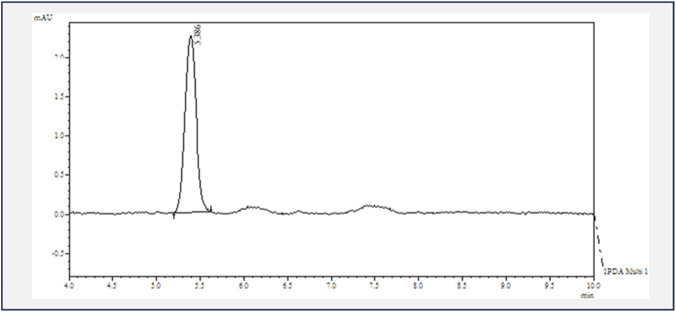
HPLC-PDA chromatogram of a representative seized powder sample under optimised chromatographic conditions; chromatographic conditions: stationary phase Hypersil GOLD™ C18 (150 × 3 mm, 3 μm); mobile phase water:methanol (96:4, v/v); flow rate 0.7 mL/min; column temperature 25 °C; detection 205 nm.

The developed HPLC method was also applied to the forensic analysis of five seized powder samples suspected of containing PGL, GBP, or a combination of both, which were the same as those previously reported (Alamri et al., 2025). The results confirmed the presence of PGL in all analysed samples (Figure 6). Regarding GBP, no peak was observed at its retention time (6.52 min) in any of the seized samples under the current HPLC-PDA conditions. This indicates that GBP was either absent or present at a concentration below the method’s LOQ (0.85 μg/mL).

Quantitative analysis of PGL further demonstrated considerable variability in the sample composition, as shown in Table 6. The assay results indicated that all five seized samples contained PGL concentrations above the reference standard value. This may indicate formulation variability, degradation, or the presence of additional components. The quantified levels fall outside the BP acceptance criteria of 95.0%–105.0% for the PGL assay of the stated amount (British Pharmacopoeia Commission, 2025a). The definitive confirmation of the presence or absence of GBP at trace levels would require a more advanced technique such as LC-MS/MS. Nevertheless, the method successfully quantified PGL in all seized samples and is suitable for routine forensic screening, provided that negative findings for GBP are interpreted conservatively. These findings demonstrate the applicability of the method for regulatory monitoring and forensic screening of seized materials.

**TABLE 6:** HPLC-PDA analysis of seized powder samples (nominal concentration: 35 μg/mL).

Powder sample	Measured concentration (µg/mL)	RSD%	Accuracy (%)
P1	38.65	0.83	110.42
P2	38.51	0.45	110.04
P3	38.64	0.76	110.40
P4	37.11	0.67	106.03
P5	37.75	1.08	107.85

### 3.5 Application of the method to dissolution testing

A 1:9 dilution of the acidic dissolution samples was implemented prior to HPLC-PDA analysis for both PGL and GBP to ensure chromatographic integrity. Aliquots of 100 µL from the 0.06 mol/L HCl (PGL) and 0.1 mol/L HCl (GBP) media were diluted with 900 µL of a water:MeOH (96:4, v/v) diluent. This step was critical to minimise the impact of the low pH on the chromatographic behaviour of both analytes. In strongly acidic media, PGL (pKa 4.2) (Kostić et al., 2014) and GBP (pKa 3.7) are predominantly ionised (Baranowska and Szeleszczuk, 2024), which can lead to inconsistent retention and peak broadening on reversed-phase columns (Ulu and Kel, 2011). The dilution effectively raised the pH of the injected sample, shifting the equilibrium of each drug towards its neutral form. This approach stabilised retention times and enabled accurate, reproducible quantification for both dissolution studies.

**FIGURE 7:**
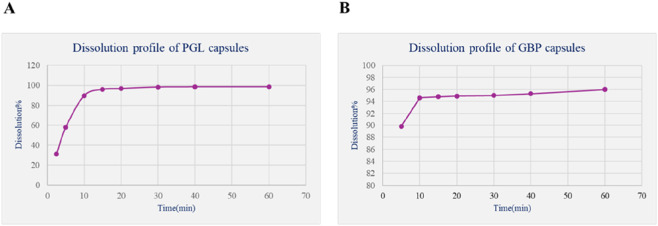
*In vitro* dissolution profiles of **(A)** 150 mg pregabalin capsules in 0.06 M HCl and **(B)** 400 mg gabapentin capsules in 0.1 M HCl.

The dissolution profiles of both PGL and GBP capsules demonstrated rapid drug release, meeting pharmacopoeial criteria. PGL achieved approximately 58% dissolution within 5 min, reaching a stable plateau of greater than 98% by 30 min. GBP exhibited an even faster initial release, with ∼90% dissolved within 5 min and a plateau of 94%–96% maintained from 10 to 60 min as summarised in Supplementary Table S4 and shown graphically in Figure 7. After applying the 10-fold dilution factor, the data confirm that both products achieve near-complete dissolution, fully complying with pharmacopoeial requirements. The stable, non-declining profiles confirm the robustness of immediate-release formulations, underscoring their high quality and consistent *in vitro* performance. Interestingly, while GBP exhibited a more rapid initial dissolution *in vitro*, PGL’s dissolution reached a greater final extent. This observation may reflect differences in formulation or physicochemical behaviour. However, *in vitro* dissolution does not necessarily predict *in vivo* performance.

Statistical comparison using the similarity factor (f_2_) (Shah et al., 1998), yielded a value of 45, confirming that the dissolution profiles of PGL and GBP are not similar (f_2_ < 50), with GBP showing faster initial release while PGL achieved higher final dissolution.

### 3.6 Comparison study

Table 7 shows comparison results of the current method against previously reported methods for the analysis of polar compounds, specifically PGL and GBP. As shown, previous methods used specialised separation conditions, which may limit their general applicability. For instance, one method required a strong cation exchange column (Udaykumar Rao et al., 2009). Other methods (Gupta et al., 2008; Ciavarella et al., 2007) used a cyano column with a buffered mobile phase. Furthermore, detection often necessitates lengthy sample preparation. Methods such as the HPLC-fluorescence assay (Ulu and Kel, 2011; Douša et al., 2010) depended on complex pre-column derivatisation to enable detection.

**TABLE 7:** Comparison of chromatographic conditions for pregabalin and gabapentin assay.

Technique	Sample preparation	Column	Mobile phase	Run time (min)	LOD µg/mL	LOQ µg/mL	Derivatization	Ref.
HPLC-UV	Extraction with methanol	Strong Cation Exchange	0.15 M NH_4_H_2_PO_4_ (pH 3.0): MeOH (60:40, v/v)	10	GBP: NR^*^	GBP: NR^*^	None	Udaykumar Rao et al. (2009)
HPLC-UV	Dissolution in diluent	Phenomenex Luna Cyano	ACN: MeOH: 20 mM KH_2_PO_4_ (pH 2.2) (5:5:90, v/v/v)	<10	GBP: 15	GBP: 50	None	Gupta et al. (2008)
HPLC-UV	Dissolution in mobile phase	Brownlee Spheri-5 Cyano	ACN: 10 mM KH_2_PO_4_/10 mM K_2_HPO_4_ (pH 6.2) (8:92, v/v)	10	GBP: 5	GBP: 18	None	Ciavarella et al. (2007)
HPLC-FL	Liquid-liquid extraction after derivatisation	C18	MeOH: H_2_O (80:20)	<10	GBP: 0.00085	GBP: 0.00255	Pre-column derivatisation	Ulu and Kel (2011)
HPLC-FL	Direct extraction	RP-8e	MeOH: 10 mM acetate buffer (pH 5.0) (15:85, v/v)	15	PGL: 4.8	PGL: 16	Post-column derivatisation	Douša et al. (2010)
HPLC-UV	Extraction with diluent (H_2_O:MeOH, 50:50 v/v)	C18	0.01 M KH_2_PO_4_ (pH 4.5): MeOH (25:75)	6	PGL: 0.08	PGL: 0.25	None	Goday et al. (2018)
HPLC-UV	Extraction with mobile phase	C18	MeOH: phosphate buffer (pH 3.0) (70:30, v/v)	10	PGL: 0.27	PGL: 0.82	None	Khanage et al. (2014)
HPLC-UV	Dissolution in mobile phase	C18	Water: MeOH (96:4, v/v)	10	PGL: 0.31 GBP: 0.25	PGL: 0.25 GBP: 0.85	None	Present study

* Not reported.

In contrast, the present method successfully overcomes these challenges by achieving satisfactory separation using a common column and a simple, unbuffered mobile phase, while simultaneously eliminating the need for derivatisation. This combination ensures robustness and accessibility for routine laboratories. Our previous UPLC-PDA method was specific to PGL and required complementary LC-MS/MS to identify GBP as an adulterant (Alamri et al., 2025). In contrast, the present method provides a unified, simultaneous assay for both gabapentinoids. This directly addresses the analytical need revealed by the earlier forensic finding. This comparison demonstrates that the current HPLC-PDA method is a more straightforward, cost-effective, and environmentally friendly alternative. By avoiding specialised columns, complex buffers, derivatisation reagents, and high solvent consumption, it is well-suited for high-throughput, routine analysis in quality control and forensic laboratories. Despite its advantages, the proposed method has certain limitations. Its sensitivity (LOD: 0.31 μg/mL for PGL, 0.25 μg/mL for GBP) is considerably lower than that of derivatisation-based fluorescence methods, such as Ulu and Kel (2011) (LOD: 0.00085 μg/mL for GBP). Therefore, the present method is not suitable for trace analysis or biological samples. Nevertheless, for routine pharmaceutical and forensic screening of bulk samples, the proposed method offers a practical, cost-effective, and environmentally friendlier alternative.

### 3.7 Greenness assessment using AGREEPrep

**FIGURE 8:**
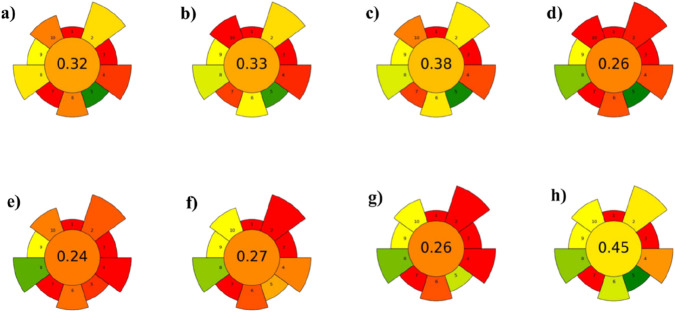
AGREEPrep pictograms of HPLC methods; **(a–g)** previous methods and **(h)** the current method.

The environmental performance of the sample preparation steps was quantitatively evaluated using the AGREEPrep metric (Wojnowski et al., 2022). As depicted in Figure 8, this assessment clearly differentiates the greenness profiles of the compared methods. Techniques that relied on derivatisation, such as pre-column (d) (Ulu and Kel, 2011) and post-column (e) (Douša et al., 2010), or that utilised high-proportion organic solvents (f) (Goday et al., 2018) and (g) (Khanage et al., 2014), achieved lower overall scores. Their performance, reflected in the AGREEPrep pictograms, stems from high penalties in key criteria: substantial consumption of hazardous solvents, considerable waste generation, and, where applicable, high energy demand. To provide a quantitative basis for the greenness comparison, organic solvent consumption per chromatographic run was calculated. Compared to the method reported by Ulu and Kel (Ulu and Kel, 2011) (4.8 mL organic solvent per run), the present method consumes 0.28 mL per run, representing a 94.2% reduction. Compared to the method reported by Khanage et al. (2014) (7.0 mL per run), the reduction is 96.0%.

The present method (h) achieved an improved AGREEPrep score compared to previously reported methods. It demonstrated good performance in solvent safety and waste minimisation. This score reflects its minimalist design: a simple dissolution in water: methanol (96:4, v/v) mobile phase, elimination of derivatisation, avoidance of specialised columns (Table 7), and reduced organic solvent consumption.

The AGREEPrep results thus provide quantitative support for the reduced solvent consumption and simplified sample preparation of the proposed method. While the method reduces organic solvent use and avoids derivatisation, its environmental advantages should be interpreted within the scope of routine pharmaceutical and forensic analysis. Therefore, the proposed approach represents a practical improvement in solvent reduction compared to some reported methods.

## 4 Conclusion

In conclusion, an environmentally favourable HPLC-PDA method has been successfully developed and validated for the simultaneous quantification of PGL and GBP. The highly aqueous mobile phase (96% water) reduces organic solvent consumption compared to selected reported methods. Through optimisation of a highly aqueous mobile phase system combined with a selective C18 column, effective baseline chromatographic separation was achieved by leveraging the subtle hydrophobicity difference conferred by the cyclohexyl ring in GBP compared to the isobutyl group in PGL. Although detection required a low wavelength (205 nm) due to the weak inherent UV absorption of these analytes, the method remains a straightforward and cost-effective solution without derivatisation. The method also demonstrated excellent resolution, precision, and accuracy, fulfilling all required validation parameters. Its practical utility was demonstrated through two key applications: quality control of commercial pharmaceutical formulations, including dissolution testing, and the forensic analysis of seized samples. In the latter role, the method effectively identified non-compliant products and showed no peak corresponding to suspected substances under the current HPLC-PDA conditions, indicating potential non-compliance with labelled content and suggesting the presence of formulation inconsistencies. Consequently, this chromatographic procedure represents a practical analytical method for routine quality control in pharmaceutical laboratories and for regulatory surveillance. It may support efforts to ensure drug quality and public health.

## Data Availability

The original contributions presented in the study are included in the article/Supplementary Material, further inquiries can be directed to the corresponding author.
