# Supplementary material for: A highly aqueous HPLC-PDA method for the assay of gabapentin and pregabalin: applications in pharmaceutical analysis, dissolution testing, and forensic screening
Source: Front Chem. 2026 Jun 26;14:1845390. doi: 10.3389/fchem.2026.1845390 (PMC13349844; doi:10.3389/fchem.2026.1845390)
Supplement: Supplementary file 1 [file Table1.docx]

**Table S1:** Full factorial design matrix and experimental results for the measured resolution (R) for all 8 runs.

| **EXP.#** | **MeOH%** | **Injection volume** | **Flow rate** | **Resolution** |
| --- | --- | --- | --- | --- |
| 1 | 4 | 10 | 0.7 | 3.303 |
| 2 | 4 | 10 | 1.5 | 2.512 |
| 3 | 4 | 20 | 0.7 | 3.101 |
| 4 | 4 | 20 | 1.5 | 2.421 |
| 5 | 6 | 10 | 0.7 | 2.865 |
| 6 | 6 | 10 | 1.5 | 2.394 |
| 7 | 6 | 20 | 0.7 | 2.606 |
| 8 | 6 | 20 | 1.5 | 2.333 |

**2³ factorial design** (three factors, each at two levels)

1. **MeOH%** → 4% and 6% (factor **A**)
2. **Injection volume** → 10 µL and 20 µL (factor **B**)
3. **Flow rate** → 0.7 mL/min and 1.5 mL/min (factor **C**)

**coded levels**

1. **A (MeOH%)**: Low = 4% (−1), High = 6% (+1)
2. **B (Inj. vol)**: Low = 10 µL (−1), High = 20 µL (+1)
3. **C (Flow rate)**: Low = 0.7 (−1), High = 1.5 (+1)

**Interaction effects**

- AB = A × B
- AC = A × C
- BC = B × C
- ABC = A × B × C

**Table S2:** 2³ full factorial design layout and experimental responses for chromatographic resolution optimisation.

| **EXP.#** | **A** | **B** | **C** | **AB** | **AC** | **BC** | **ABC** | **Resolution(R)** |
| --- | --- | --- | --- | --- | --- | --- | --- | --- |
| 1 | −1 | −1 | −1 | +1 | +1 | +1 | −1 | 3.303 |
| 2 | −1 | −1 | +1 | +1 | −1 | −1 | +1 | 2.512 |
| 3 | −1 | +1 | −1 | −1 | +1 | −1 | +1 | 3.101 |
| 4 | −1 | +1 | +1 | −1 | −1 | +1 | −1 | 2.421 |
| 5 | +1 | −1 | −1 | −1 | −1 | +1 | +1 | 2.865 |
| 6 | +1 | −1 | +1 | −1 | +1 | −1 | −1 | 2.394 |
| 7 | +1 | +1 | −1 | +1 | −1 | −1 | −1 | 2.606 |
| 8 | +1 | +1 | +1 | +1 | +1 | +1 | +1 | 2.333 |

**Table S3:** Calculated main and interaction effects from the 2³ full factorial design for chromatographic resolution.

| Effect Type | Factor/Interaction | Avg. at High Level | Avg. at Low Level | Effect Value |
| --- | --- | --- | --- | --- |
| Main | A (MeOH%) | 2.5495 | 2.83425 | −0.28475 |
|  | B (Inj. Vol) | 2.61525 | 2.7685 | −0.15325 |
|  | C (Flow Rate) | 2.415 | 2.96875 | −0.55375 |
| Interaction | AB | 2.6885 | 2.69525 | −0.00675 |
|  | AC | 2.78275 | 2.601 | +0.18175 |
|  | BC | 2.7305 | 2.65325 | +0.07725 |
|  | ABC | 2.70275 | 2.681 | +0.02175 |

**Table S4:** In vitro dissolution profiles of pregabalin and gabapentin capsules

| **Analyte (Dose, Medium)** | **Time (min)** | **Corrected % Dissolved**  **(Mean, n=6)** |
| --- | --- | --- |
| Pregabalin  (150 mg, 0.06 M HCl) | 2.5 | 31.0% |
|  | 5 | 58.0% |
|  | 10 | 89.7% |
|  | 15 | 95.94% |
|  | 20 | 96.8% |
|  | 30 | 98.3% |
|  | 40 | 98.5% |
|  | 60 | 98.6% |
| Gabapentin  (400 mg, 0.1 M HCl) | 5 | 89.8% |
|  | 10 | 94.6% |
|  | 15 | 94.8% |
|  | 20 | 94.9% |
|  | 30 | 95.0% |
|  | 40 | 95.3% |
|  | 60 | 96% |
